# Fibroblast activation protein inhibitor (FAPI) PET imaging in pulmonary fibrosis: pathophysiology, clinical utility, and emerging theranostic applications

**DOI:** 10.1186/s12931-026-03661-y

**Published:** 2026-04-14

**Authors:** Ali Al-waqeerah, Huanyu He, Ahmed Bashah, Eslam Ghaleb, Nan Wang, Lili Gao

**Affiliations:** 1 Department of Respiratory Medicine, The First Affiliated Hospital of Dalian Medical University, Dalian, 116011 People’s Republic of China; 2 Department of Stomatology, The First Affiliated Hospital of Dalian Medical University, Dalian, 116011 People’s Republic of China; 3 College of Basic Medical Science, Dalian Medical University, Dalian, 116044 People’s Republic of China

**Keywords:** Fibroblast, Fibroblast Activation Protein Inhibitor (FAPI), Positron Emission Tomography (PET), Pulmonary Fibrosis, Fibroblast Activation Protein, Theranostics

## Abstract

Pulmonary fibrosis arises from a dysregulated wound-healing response in which activated fibroblasts drive excessive extracellular matrix deposition and irreversible lung architectural distortion. Conventional high-resolution computed tomography (HRCT) detects largely established structural damage, limiting opportunities for early intervention. The fibroblast activation protein (FAP), a membrane-bound serine protease expressed on activated fibroblasts, presents a potential molecular target for imaging active fibrogenesis. The advent of fibroblast activation protein inhibitor (FAPI)–based radiotracers for positron emission tomography (PET) has been developed to visualize FAP expression in vivo. This review traces the translational continuum from FAP biology through radiotracer development to clinical application of FAPI-PET/CT in interstitial lung diseases, summarising preclinical and emerging human studies investigating FAPI PET/CT in interstitial lung diseases, highlighting preliminary evidence for the detection of early fibroblast activation, potential risk stratification, and monitoring of therapy response. We also discuss the functional heterogeneity of FAP-expressing fibroblast subsets and the interpretive challenges this poses for FAPI imaging. The review also considers early translational work on FAPI-based theranostics, combining diagnostic imaging with fibroblast-targeted radioligand approaches. While FAPI-PET/CT shows promise as a tool for characterizing fibrogenic activity, current evidence remains limited, and prospective studies are needed to clarify its clinical utility and potential role in guiding personalized interventions.

**Graphical Abstract:**

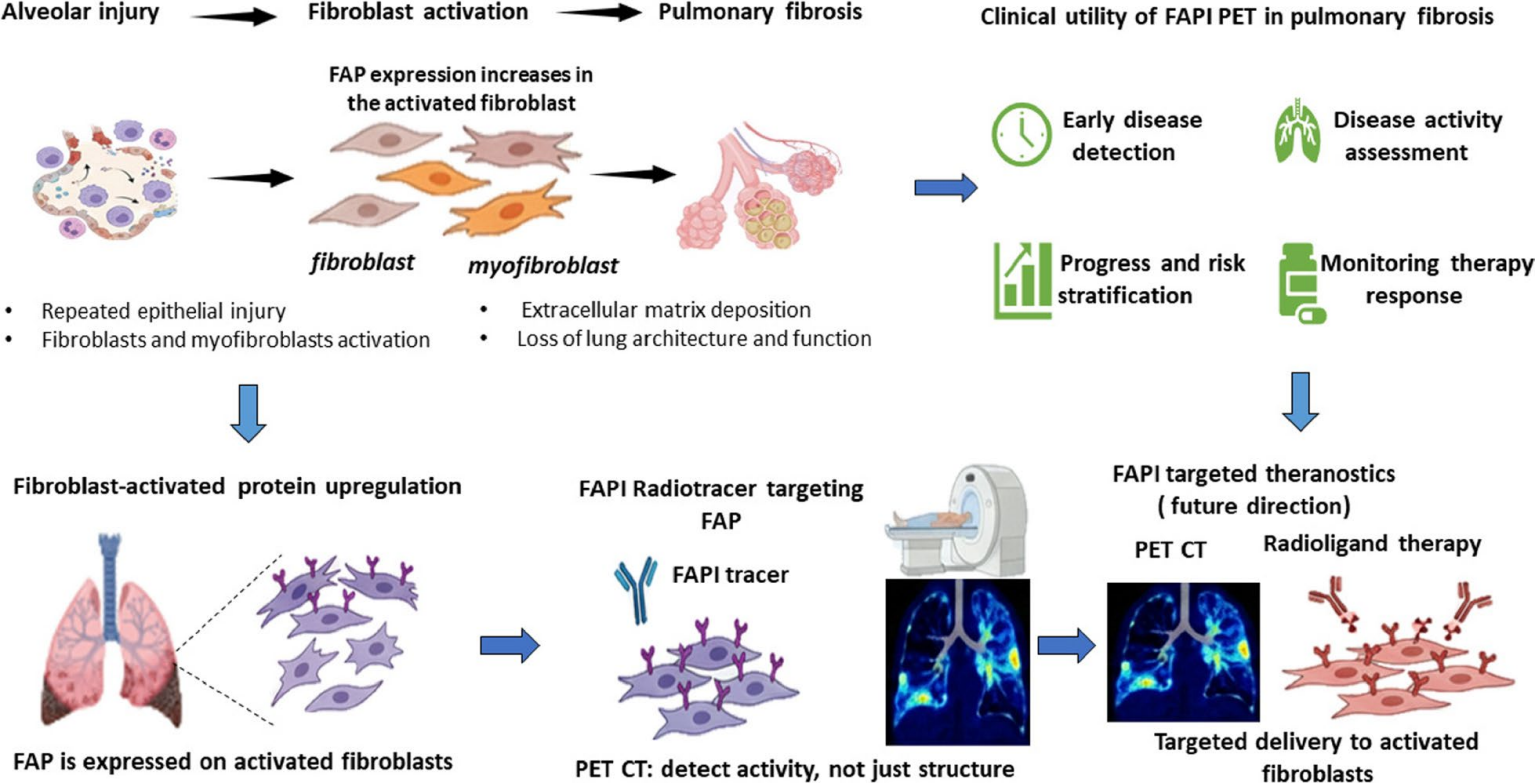

## Introduction

Fibrosis represents a key component of the wound-healing response and is a conserved biological process observed across multiple organ systems [1]. While it serves an essential role in preserving tissue architecture and maintaining organ function in the acute phase following injury, dysregulation of this process can result in pathological fibrosis [2, 3]. This maladaptive response is characterized by excessive extracellular matrix deposition, tissue stiffening, and ultimately, progressive impairment of organ function [4].

In the lung, Pulmonary fibrosis is a pathologic process underlying nearly all chronic interstitial lung diseases (ILDs) and thus a target of choice for both imaging diagnosis and therapy [5]. The success of these approaches, however, depends on the ability to detect and manage aberrant fibrotic activity at an early stage, while preserving the lung’s vital reparative processes. This ambivalent function of fibrosis is better appreciated in acute lung injury, where early fibrotic response serves a beneficial purpose by stabilizing alveolar architecture and limiting secondary tissue injury. Yet, when this reaction becomes dysregulated or overproduced, it may cause chronic fibrotic remodeling with distortion of lung parenchymal structure, compromised gas exchange, and ultimately progression to respiratory failure in conditions such as idiopathic pulmonary fibrosis (IPF) and other forms of fibrotic ILD [6, 7]. A better understanding of the timing and regulation of appropriate versus pathological fibrotic responses in the lung is crucial for preventing progression to irreversible pulmonary fibrosis while preserving the protective aspects of tissue repair.

Non-invasive detection of pulmonary fibrosis remains critically important. High-resolution CT (HRCT) continues to be the clinical gold standard for visualizing established fibrotic changes [8, 9]. Quantitative CT (Q‑CT) enhances this by providing more objective and reproducible assessments of disease extent and progression [10]. MRI techniques—particularly T1 mapping—have demonstrated the ability to detect early interstitial changes( like inflammation and edema), potentially before CT shows structural abnormalities [11]. While conventional imaging excels at identifying irreversible remodeling, its spatial resolution limits its ability to detect early fibrosis in small airways, the pleura, or pulmonary vasculature. These methods play crucial roles in diagnosis and risk stratification across interstitial lung diseases; however, their limitations underscore the need for improved imaging and analytical tools [8, 12].

Novel molecular positron emission tomography (PET) radiotracers, including radiolabeled fibroblast activation protein inhibitors (FAPI), have been developed with the aim of imaging fibroblast activation and fibroproliferative processes [13, 14]. FAPI imaging has gained significant research interest and growing clinical exploration for examining fibroblast activation and fibrotic processes throughout various organ systems, expanding upon its proven utility in visualizing cancer-associated fibroblasts [15]. More recently, there has been a growing exploration of its potential application in fibrotic lung disease. However, this field remains at an early stage, and many questions relating to specificity, reproducibility, and prognostic value are still unresolved. Fibroblast activation protein inhibitor (FAPI) PET imaging has emerged as a promising molecular imaging approach under investigation for assessing fibroblast activity in pulmonary fibrosis [15, 16].

This review provides an overview of the current status of FAPI PET imaging in preclinical and early clinical pulmonary fibrosis, emphasizing its potential clinical applications and outlining key directions for future research. We begin by summarizing the underlying pathophysiology of fibrotic lung diseases, highlighting the central role and phenotypic heterogeneity of FAP-positive activated fibroblasts in extracellular matrix deposition and disease progression. The application of FAPI tracers in visualizing these processes is discussed in the context of both preclinical and emerging clinical studies. We then critically evaluate the existing literature on FAPI imaging in pulmonary fibrosis, addressing current technical challenges and inter-subtype variability. Finally, we explore how this novel imaging approach could enhance early diagnosis, improve disease monitoring, and facilitate precision treatment strategies in the management of interstitial lung diseases. However, most evidence supporting FAPI PET in pulmonary fibrosis is still preliminary, with human data largely derived from small pilot cohorts rather than large controlled studies.

## Fibroblast activation and its contribution to fibrosis

Pulmonary fibrosis results from excessive deposition of extracellular matrix (ECM), primarily composed of fibrillar collagens, within the lung interstitium [17, 18]. The cross-linking of these fibers decreases lung compliance and elasticity, thereby impairing gas exchange and lung function [19, 20]. Pulmonary fibrosis progresses along a continuum, from early, potentially reversible inflammation or fibrosis to established, irreversible scarring [21, 22]. Acute injuries like post-ARDS or early hypersensitivity pneumonitis may resolve or stabilize once the underlying trigger is removed [6, 23]. In contrast, chronic fibrosing diseases such as idiopathic pulmonary fibrosis (IPF) and connective tissue disease-related ILD lead to permanent architectural distortion, primarily characterized by excessive extracellular matrix deposition [24, 25]. While antifibrotic medications like pirfenidone and nintedanib can slow functional decline in diseases like IPF, they do not reverse established fibrosis [26, 27].

Activated fibroblasts are considered central effector cells in pulmonary fibrosis, responsible for synthesizing collagen and other extracellular matrix (ECM) components within the lung interstitium [28–30]. These fibroblasts or myofibroblast cells may originate from multiple sources, including resident fibroblasts, epithelial cells undergoing epithelial-to-mesenchymal transition (EMT), endothelial cells through endothelial-to-mesenchymal transition (EndMT), and circulating fibrocytes recruited from the bone marrow [31–35]. In their quiescent state, fibroblasts maintain tissue homeostasis by regulating ECM turnover and monitoring lung architecture [36, 37]. Upon injury or chronic inflammation, fibroblasts become activated and can differentiate into myofibroblasts, a heterogeneous population that produces excessive ECM, leading to lung stiffening and impaired gas exchange, contributing significantly to disease progression in pulmonary fibrosis [38, 39].

**Fig. 1:**
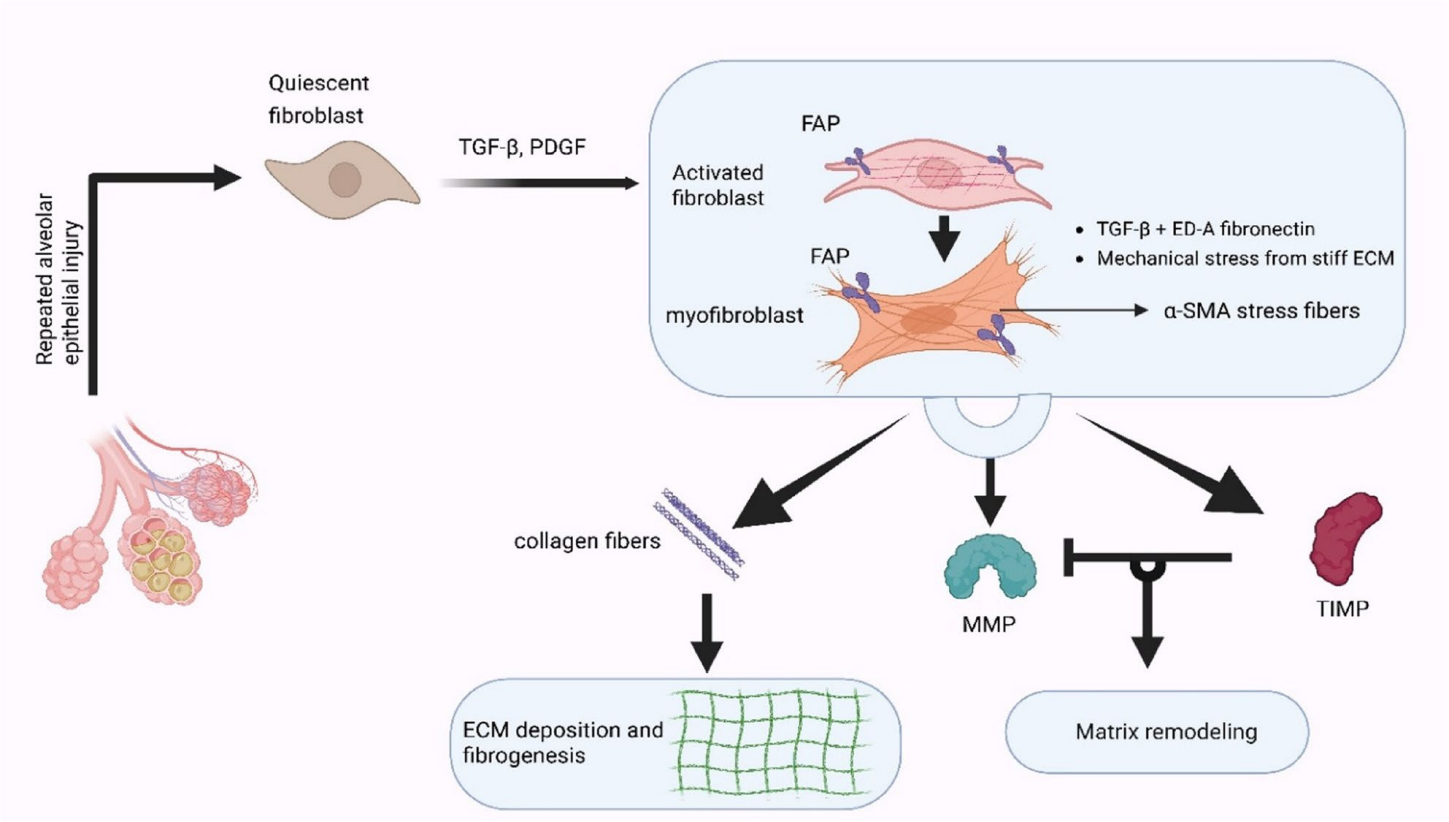
Cascade of lung fibrosis formation regulated by activated fibroblasts. Following repeated alveolar epithelial injury, quiescent fibroblasts are activated and differentiate into activated fibroblasts under the influence of cytokines such as TGF-β and PDGF. Continued cytokine signaling, together with modulators such as ED A-fibronectin and mechanical stress, promotes their maturation into fully differentiated myofibroblasts. These myofibroblasts are characterized by thick, interconnected α-SMA–positive actin fibers and the formation of mature, stable adhesion plaques at membrane–ECM junctions, leading to increased contractile strength. Abbreviations: TGF-β, transforming growth factor-beta; PDGF, platelet-derived growth factor; ECM, extracellular matrix; MMP, matrix metalloproteinases; TIMP, tissue inhibitor of matrix metalloproteinases; α-SMA, alpha-smooth muscle actin; EDA, extra domain A fibronectin

In pulmonary fibrosis, activated lung fibroblasts drive pathological fibrogenesis, as observed in fibrosis affecting other organs [40, 41]. Initially, fibroblasts become activated fibroblasts, gaining proliferative and migratory capacities in response to cytokines and growth factors, such as TGF-β, PDGF [42, 43]. These cells infiltrate sites of epithelial injury and begin depositing collagen-rich ECM [28, 44]. Sustained TGF-β signaling, combined with ECM-derived cues (e.g., ED A fibronectin) and mechanical stress from a stiffening matrix, then drives maturation into contractile myofibroblasts. This terminal stage is characterized by Alpha smooth muscle actin (α-SMA) stress fibers and fibronexus adhesions, which further stiffen and contract lung tissue [7, 45, 46]. Various cell types in the fibrotic lung microenvironment secrete multiple matrix metalloproteinases (MMPs), notably MMP-2, -3, -8, -11, -12, and − 28, all of which are upregulated and contribute to profibrotic remodeling. Concurrently, tissue inhibitors of metalloproteinases (TIMPs 1 to 4) are also overexpressed; among them, TIMP‑2 is particularly enriched in fibroblast foci. This imbalance between MMPs and TIMPs shifts the proteolytic environment toward reduced ECM degradation, thereby promoting persistent collagen accumulation, as shown in Fig. 1.

Interestingly, despite their classical ECM-degrading roles, certain MMPs, especially MMP‑3 and MMP‑7, exert paradoxical profibrotic effects by activating Wnt/β‑catenin signaling, cleaving E‑cadherin, and facilitating epithelial-to-mesenchymal transition (EMT), thereby amplifying fibrogenesis [47–49]. Thus, the interplay of collagen deposition, impaired degradation, and ECM remodeling, potentially augmented by processes such as EMT, drives the relentless scarring observed in pulmonary fibrosis [28, 50, 51].

## Fibroblast activation protein

Fibroblast activation protein (FAP) is a surface-bound glycoprotein consisting of 760 amino acids, predominantly found on activated fibroblasts rather than their quiescent counterparts [52, 53]. Although absent in the vast majority of adult tissues under normal physiological conditions, with very low levels reported in specific tissues like endometrial cells [54] and pancreatic alpha cells [55], FAP plays a physiological role during embryogenesis and organ development, reflecting its involvement in regulated tissue remodeling processes [52, 56, 57]. First characterized in 1994, FAP exhibits both dipeptidyl peptidase and endopeptidase enzymatic functions, distinguishing it from the more widely expressed dipeptidyl peptidase IV (DPP IV or CD26), primarily due to its unique endopeptidase activity [52, 58]. This selective expression pattern makes FAP a valuable biomarker of fibroblast activation and an indirect indicator of ongoing fibrotic remodeling in various disease contexts [59, 60]. FAP-expressing fibroblasts are key players observed across multiple pathological conditions involving fibrosis, such as wound healing, hepatic fibrosis, and pulmonary fibrosis, where they significantly contribute to the excessive deposition of extracellular matrix among various contributing cell types and pathways [60, 61]. Moreover, in the tumor microenvironment, FAP is a defining marker of cancer-associated fibroblasts (CAFs). Its protease activity supports cell invasion and migration, and its expression is strongly and frequently associated with tumor progression and a poor prognosis, highlighting its potential as a therapeutic target in oncology [62–64]. While the dominant role of FAP+ CAFs is pro-tumorigenic, context-dependent complexities in their function exist [65].

## Fibroblast activation protein inhibitor radiotracers

The development of fibroblast activation protein (FAP)-targeted radiotracers is based on quinoline-derived inhibitors conjugated to chelators for radiolabeling. Early-generation compounds, such as FAPI-02 and FAPI-04, utilized DOTA (1,4,7,10-tetraazacyclododecane-1,4,7,10-tetraacetic acid) for complexation with isotopes like ⁶⁸Ga or ¹⁸F-AlF [66–68]. These tracers demonstrated promising clinical potential, exhibiting excellent serum stability, high binding affinity, and favorable target-to-background imaging ratios. However, they were associated with significant hepatobiliary excretion, which may compromise imaging quality [67, 69, 70]. The subsequent development of FAPI-46, still utilizing DOTA, resulted in increased tumor uptake and comparable healthy-organ uptake (leading to improved tumor-to-background ratios) relative to FAPI-04 [71–73]. To address limitations of DOTA compatibility with ¹⁸F-AlF complexation, specifically slower kinetics and lower specific activity, researchers turned to NOTA (1,4,7-triazacyclononane-1,4,7-triacetic acid), a smaller chelating agent more suitable for ¹⁸F-AlF complexation. This shift resulted in the generation of FAPI-74 [74].

Recent literature confirms that NOTA-based FAPI derivatives, such as FAPI-74 and Al¹⁸F-NOTA-FAPI, enable efficient ¹⁸F-AlF labeling under mild conditions and yield higher radiochemical purity and specific activity [74, 75]. Beyond NOTA, alternative chelators such as H₃RESCA have been reported for Al¹⁸F labeling, achieving high yields and in vivo stability, thereby expanding the toolkit for clinical translation [76]. Meanwhile, DATA5m and AAZTA chelators have emerged in exploratory FAP-targeting studies, demonstrating efficient ⁶⁸Ga labeling and favorable biodistribution, though their use in ¹⁸F-FAPI tracers remains limited [77, 78]. In parallel, ONCOFAP emerged as a next-generation FAP-targeting ligand with high binding affinity, potentially enhanced specificity, and excellent target-to-background contrast. Notably, ONCOFAP is amenable to conjugation with both DOTA and NOTA, enabling radiolabeling with ⁶⁸Ga and ¹⁸F-AlF for diagnostic imaging. Furthermore, the closely related therapeutic agent ONCOFAP-23 can be labeled with ¹⁷⁷Lu, enabling investigational theranostic applications in conjunction with diagnostic purposes [79–81]. Additionally, ⁹⁹ᵐTc-labeled tracers, such as ⁹⁹ᵐTc-HFAPI, are being developed for SPECT imaging, thereby extending FAPI-based diagnostics to regions lacking PET infrastructure [82].

At present, a clear stratification of FAPI tracers according to their relative clinical utility in pulmonary fibrosis cannot be definitively established. Most available data are derived from early-phase, heterogeneous studies, with limited direct comparisons between tracers in interstitial lung disease. Although newer compounds, such as FAPI-46 and FAPI-74, exhibit improved pharmacokinetic properties and labeling flexibility, their comparative performance in fibrotic lung disease remains insufficiently validated. Therefore, current evidence does not support a definitive hierarchy among FAPI tracers, and they should be considered broadly comparable pending further head-to-head and disease-specific investigations.

## FAPI imaging in interstitial lung disease

The nascent literature supporting the potential role of FAPI radiotracers in interstitial lung disease (ILD) is expanding, with evidence derived from both preclinical models and initial human studies. The following sections stratify this evidence to provide a clearer logical framework. However, current data remain preliminary, and interpretation must account for small sample sizes, heterogeneous patient cohorts, and limited longitudinal validation.

### Preclinical evidence from murine models

Preclinical studies using established mouse models of pulmonary fibrosis, most commonly bleomycin-induced and, to a lesser extent, paraquat-induced injury, have been instrumental in validating FAPI PET. These studies consistently demonstrate that FAP-targeted PET tracers can detect molecular changes associated with fibrogenesis earlier than structural abnormalities become apparent on CT.

For instance, in bleomycin models, FAPI lung uptake increases by day 7, whereas CT abnormalities are typically visible around day 14 [83]. Similarly, in paraquat-induced models, Al¹⁸F-NODA-FAPI-04 uptake is elevated in fibrotic regions before clear CT changes are observed [84]. In preclinical murine models of lung fibrosis, pulmonary FAPI uptake paralleled collagen deposition and FAP expression, with peak accumulation occurring later than FDG, suggesting, but not definitively proving, that FAPI PET is a potential marker of active fibrogenesis, as shown in Fig. 2 from Ji et al., Pharmaceuticals 2024, 17, 726, under CC BY 4.0 [85]. Across these models, tracer uptake (e.g., ⁶⁸Ga-FAPI-46, ¹⁸F-FAPI-74, ¹⁸F-FAPI-42) correlates closely with markers of active fibrogenesis: FAP expression (via IHC), collagen deposition, and histologic severity of fibrosis.

**Fig. 2:**
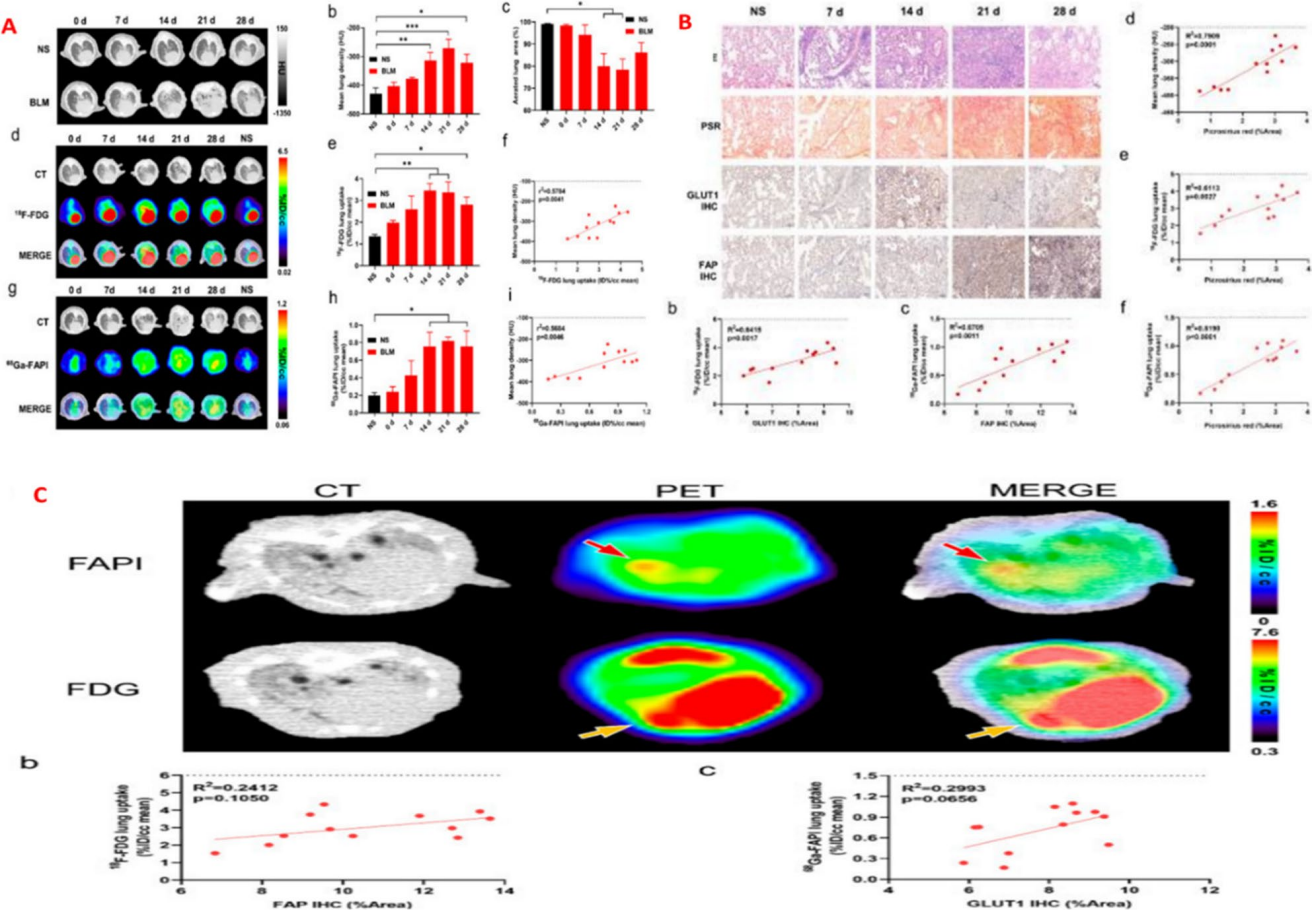
Example of [⁶⁸Ga] FAPI PET enables specific detection and monitoring of pulmonary fibrosis progression in a bleomycin-induced mouse model, compared to [¹⁸F] FDG PET and CT. **A** In the red label, temporal imaging revealed an earlier peak of [¹⁸F] FDG uptake (Day 14) and a later peak of [⁶⁸Ga] FAPI uptake and CT lung density (Day 21), reflecting distinct pathological phases. **B** In the red label, [⁶⁸Ga] FAPI uptake strongly correlated with collagen deposition (PSR staining, r² = 0.819), while [¹⁸F] FDG correlated with GLUT1 expression. **C** In the red label, at Day 21, [⁶⁸Ga] FAPI and [¹⁸F] FDG highlighted distinct lesion foci within the same CT-detected consolidations, corresponding to FAP⁺ fibroblasts and GLUT1⁺ inflammatory cells, respectively. Reproduced from Ji et al. [⁶⁸Ga] FAPI PET for imaging and treatment monitoring in a preclinical model of pulmonary fibrosis: comparison to [¹⁸F] FDG PET and CT

Direct comparisons with FDG suggest differing uptake patterns; however, clear evidence of superior specificity is still limited and may vary by model and inflammatory context [86]. Importantly, FAP can also be upregulated in certain inflammatory or reparative remodeling settings, which means uptake may not exclusively represent progressive fibrosis [87]. Significantly, these tracers have shown utility in monitoring therapeutic response. For example, pirfenidone treatment in bleomycin models results in decreased ⁶⁸Ga-FAPI uptake, concurrent with histologic improvement [85]. Emerging data with Nintedanib and ⁶⁸Ga-FAPI-04 also demonstrate dynamic uptake changes over time with treatment [88]. A summary of these preclinical findings is presented in Table 1 [84, 85, 88–91].

**Table 1:** Overview of the included animal studies

Author (Year)	Model	Tracer	Measures of fibrosis	Peak Uptake / Timepoint	Key Findings
Rosenkrans et al. (2022) [89]	Bleomycin-induced pulmonary fibrosis	⁶⁸Ga-FAPI-46	Histology (Masson’s trichrome stain, IHC for FAP-α expression), CT.	Increased from Day 7 to 14.	Tracer Detected fibrotic activity earlier (day 7) than CT (day 14); uptake correlated with FAP expression, collagen content, and histologic severity.
Lavis et al. (2023) [90]	Bleomycin; translational IPF cohort	¹⁸F-FAPI-74	Histology (Masson’s trichrome stain and IHC for FAP-α expression), High-performance liquid chromatography, CT	Day 10 peak; declined with Nintedanib	BALF FAPα levels paralleled imaging uptake, an early predictor of fibrogenic progression.
Zhang et al. (2024) [91]	Paraquat-induced lung injury	¹⁸F-FAPI-42	Histology (HE, Masson’s trichrome stain, fluorescent probe staining for FAP-α), CT.	Day 14	Early uptake in CT-negative zones signaled pre-structural fibrosis; it correlated with collagen staining and perfusion loss.
Song et al. (2023) [84]	Paraquat-induced fibrosis	Al¹⁸F-NODA-FAPI-04	Histology (Masson’s trichrome stain and IHC for FAP-α expression), Western Blot, Low-dose CT		FAPI uptake was higher in PQ mice, matching histology and Western blot.
Ji et al. (2024) [85]	Bleomycin model ± pirfenidone	⁶⁸Ga-FAPI / ¹⁸F-FDG	Histology (HE, picrosirius red, and IHC for FAP-α and GLUT-1 expression), CT.	FDG peak Day 14; FAPI peak Day 21	Distinct inflammatory vs. fibrotic phases; FAPI decreased with pirfenidone treatment.
Sun et al. (2025) [88]	Bleomycin-induced fibrosis ± nintedanib	⁶⁸Ga-FAPI-04	Histology (HE and FAP immunohistochemistry (IHC) staining), CT.	Week 4	The tracer tracked fibrosis progression and correlated with FAP expression; nintedanib reduced SUVRs, indicating an antifibrotic response.

Table 1. Summary of murine studies utilizing FAPI imaging

*Abbreviations*: *BLM* bleomycin, *BALF* bronchoalveolar lavage fluid, *CT* computed tomography, *FAP* fibroblast activation protein, *FDG* fluorodeoxyglucose, *IPF* idiopathic pulmonary fibrosis, *PET* positron emission tomography, *PQ* paraquat, *SUVR* standardized uptake value ratio

### Emerging clinical evidence in human ILD

Emerging clinical studies have explored FAP-targeted PET imaging, primarily using ⁶⁸Ga-FAPI-04 and ⁶⁸Ga-FAPI-46, in various interstitial lung diseases (ILDs), including idiopathic pulmonary fibrosis (IPF) [92], connective tissue disease-associated ILD (CTD-ILD) [93, 94], systemic sclerosis-related ILD (SSc-ILD) [95], and idiopathic inflammatory myopathy-associated ILD (IIM-ILD) [96]. These studies are generally small, single-center, and exploratory, and most lack robust histopathological confirmation or long-term outcome data.

These studies consistently report increased FAPI tracer uptake in affected lung regions versus healthy controls. Several studies show correlations between tracer uptake and measures of disease severity, such as forced vital capacity (FVC), diffusing capacity of the lung for carbon monoxide (DLCO), and the extent of fibrosis on high-resolution computed tomography (HRCT) [94, 96]. Compared with conventional ¹⁸F-FDG PET/CT, preliminary data suggest FAPI-PET/CT may offer improved image contrast in fibrotic lung and a different uptake profile that could better reflect fibroblast activation than glucose metabolism [85].

**Fig. 3:**
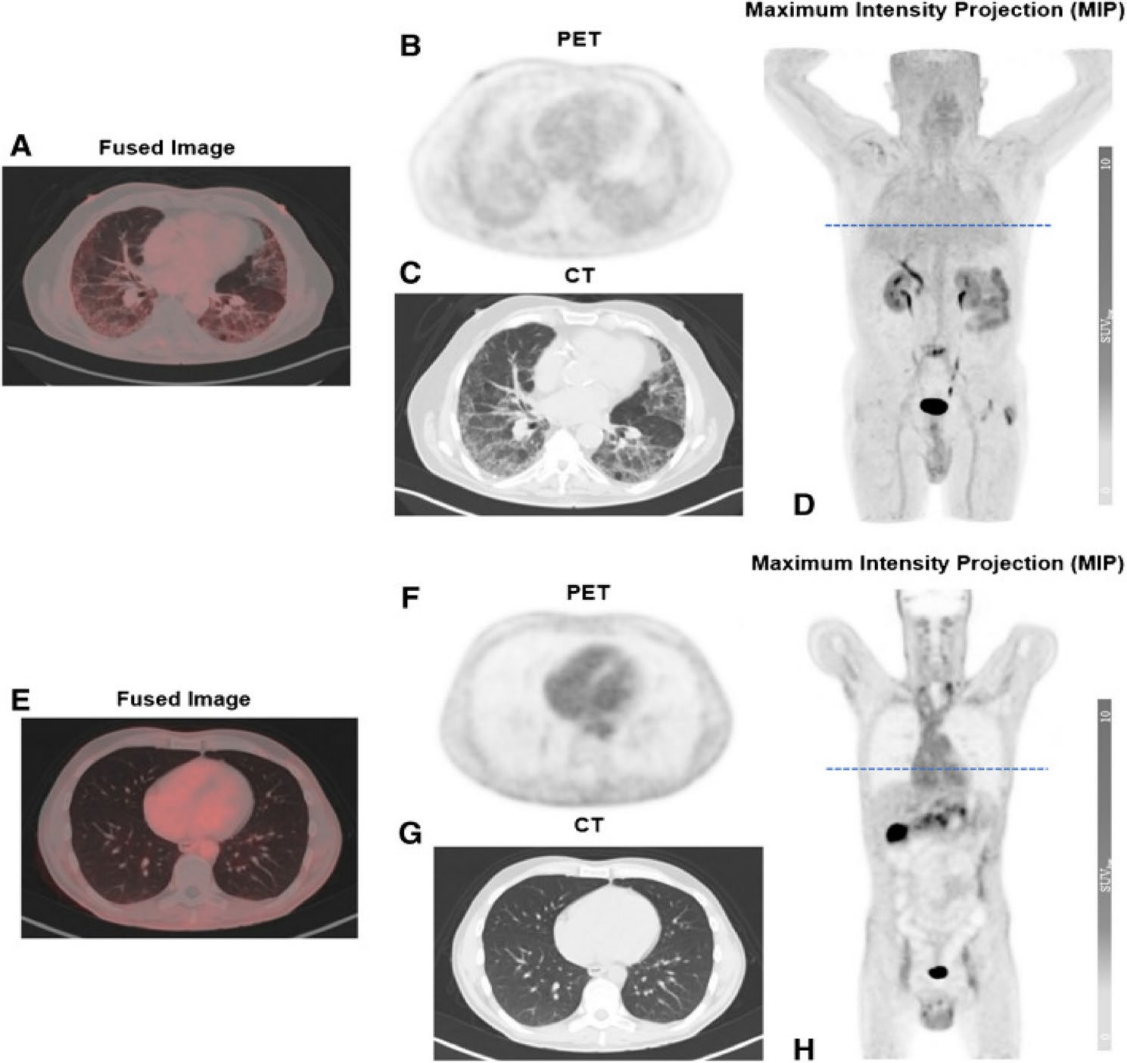
Examples of [¹⁸F] FAPI-74 uptake in idiopathic pulmonary fibrosis. **A**–**D**: Representative [¹⁸F] FAPI-74 PET/CT images of a 68-year-old male patient with idiopathic pulmonary fibrosis showing axial fusion (**A**), PET (**B**), CT (**C**), and maximum intensity projection (**D**). Positive tracer uptake is present in concordant fibrotic regions but absent in non-fibrotic lung areas. **E**–**H**: Corresponding images of a 56-year-old male patient with pancreatic adenocarcinoma and no known lung disease as control (**E**: axial fusion, **F**: PET, **G**: CT, **H**: maximum intensity projection). Reproduced from Mori et al. Initial results with [¹⁸F] FAPI-74 PET/CT in idiopathic pulmonary fibrosis

For instance, ^18^F-FAPI-74 PET/CT scans have demonstrated increased tracer uptake in fibrotic lung regions, with uptake levels correlating with disease severity and pulmonary function test results Fig. 3 [92]. Limited histopathological correlation has been reported, but comprehensive quantitative validation is still lacking. For example, Hotta et al. (2024) reported FAPI uptake in areas showing FAP expression on immunohistochemistry in explanted ILD lungs; however, the number of samples was small, and the correlation was qualitative rather than fully quantitative, so statements implying definitive histopathologic validation should be made cautiously [93]. Furthermore, recent clinical studies have expanded the utility of FAPI-PET across ILD subtypes. In idiopathic inflammatory myopathy-associated ILD (IIM-ILD), ⁶⁸Ga-FAPI PET demonstrated increased pulmonary uptake associated with disease activity and showed a potential association with subsequent functional decline, suggesting potential as a biomarker of progressive fibrosis [97]. Finally, the first-in-human study of ¹⁸F-FAPI-74 in idiopathic pulmonary fibrosis (IPF) confirmed high uptake in fibrotic lung regions with good lesion-to-background contrast, supporting the feasibility rather than establishing diagnostic accuracy [92].

Distinct uptake patterns between ILD subtypes have been reported in small series and may aid differential diagnosis, though these findings remain exploratory and require multicenter validation. Early longitudinal data indicate that baseline or changing FAPI uptake may predict progression or reflect response to antifibrotic therapy. However, standardized quantification methods and larger prospective cohorts are needed before clinical implementation. These findings are summarized in Table 2 [60, 82, 84, 92, 93, 95, 96, 98, 99].

**Table 2:** Overview of the included human studies

Author (Year)	Patient Cohort (*n*)	Tracer	Imaging Modality	Comparative / Correlative Measures	Key Findings
Yang et al. (2023) [60, 61]	ILD = 83; Controls = 8	⁶⁸Ga-FAPI-04	PET/CT	HRCT; FVC; DLCO.	FAPI Uptake (SUVtotal) correlated with fibrotic burden and lung-function decline; distinguished IPF vs. non-IPF patterns.
Bergmann et al. (2021) [95]	SSc-ILD = 21; Controls = 21	⁶⁸Ga-FAPI-04	PET/CT	HRCT; FVC	Increased FAPI uptake predicted ILD progression independently of HRCT extent and baseline FVC, and sequential scans showed uptake changes concordant with responses to nintedanib.
Kastrati et al. (2024) [96]	IIM-ILD = 14; Controls = 21	⁶⁸Ga-DATA5m.SA.FAPI	PET/CT	PFTs; HRCT	FAPI uptake strongly correlated with disease extent on HRCT and NYHA symptom severity, and inversely with FVC and DLCO.
Röhrich et al. (2022) [98]	ILD + suspected lung cancer = 15	⁶⁸Ga-FAPI-46	PET/CT	Static, dynamic imaging, and CT-based fibrosis index.	Both fILD lesions and lung cancer showed markedly increased FAPI uptake, with distinct time–activity curves; uptake positively correlated with the CT-based fibrosis index.
Hotta et al. (2024) [93]	ILD (IPF, RA, NSIP) = 4 (explanted lungs)	⁶⁸Ga-FAPI-46	PET/CT	FAPI uptake and FAP IHC correlation	FAPI uptake localized to CT-defined fibrotic areas, with FAP staining positive in fibroblastic foci, showing a positive correlation with FAP expression scores.
Bahtoue et al. (2025) [99]	ILD (IPF, CTD, NSIP, IPAF) = 20, controls = 10	⁶⁸Ga-FAPI-46	PET/CT, and 99 m Tc-MIBI SPECT/CT	HRCT, FAPI PET, and MIBI scans	FAPI and MIBI scans were each positive in 60% of ILD patients, with some overlap and discordance; FAPI uptake correlated positively with HRCT fibrosis scores and differed significantly from controls.
Mori et al. (2024) [14, 92]	IPF = 8; Controls = 6	¹⁸F-FAPI-74	PET/CT	HRCT, FAV, HF scale DLCO; FVC.	Positive correlation between the fibrotic active volume, the Hounsfield scale, as well as the vital and diffusing capacity of the lung
Liu et al. (2023) [82]	IPF = 11	⁹⁹mTc-HFAPI	SPECT	HRCT; PFTs	IPF patients showed increased ⁹⁹ᵐTc-HFAPI uptake in fibrotic lung regions, correlating with HRCT stage and moderately with PFT results.
Song et al. (2023) [84]	PQ poisoning = 2	Al¹⁸F-NODA-FAPI-04	PET/CT	Histology; IHC, WB	FAPI contributed to PQ-induced fibrosis, and FAPI PET/CT detected lung fibrogenesis.

Table 2. Summary of human studies utilizing FAPI PET imaging

*Abbreviations*: *CTD* connective tissue disease, *DLCO* diffusing capacity of the lungs for carbon monoxide, *FAV* fibrotic active volume, *FAP* fibroblast activation protein, *FVC* forced vital capacity, *HF* Hounsfield (scale), *HRCT* high-resolution computed tomography, *IHC* immunohistochemistry, *IIM-ILD* idiopathic inflammatory myopathy-associated interstitial lung disease, *ILD* interstitial lung disease, *IPAF* interstitial pneumonia with autoimmune features, *IPF* idiopathic pulmonary fibrosis, *MIBI* methoxyisobutylisonitrile, *NSIP* nonspecific interstitial pneumonia, *NYHA* New York Heart Association, *PET* positron emission tomography, *PFTs* pulmonary function tests, *PQ* paraquat, *RA* rheumatoid arthritis, *SPECT* single-photon emission computed tomography, *SSc-ILD* systemic sclerosis-associated interstitial lung disease, *SUV* standardized uptake value, *WB* western blot

## Other fibrosis radiotracers

Beyond FAPI-based imaging, several other radiotracers targeting fibrotic pathways are under investigation, although current data remain preliminary. A particularly important emerging approach is collagen-targeted molecular imaging using [⁶⁸Ga]-CBP8, a radiotracer that directly binds to type I collagen, a major structural component of fibrotic extracellular matrix. Preclinical studies demonstrated high specificity for fibrotic tissue, with tracer uptake strongly correlating with lung collagen content in animal models of pulmonary fibrosis [100]. First-in-human studies confirmed that ⁶⁸Ga-CBP8 is safe, with favorable pharmacokinetics, rapid renal clearance, and low background lung uptake, while showing significantly increased pulmonary signal in patients with idiopathic pulmonary fibrosis compared to healthy controls [101, 102]. Longitudinal imaging studies further suggest that CBP8 may be useful for monitoring therapeutic response, with changes in tracer uptake reflecting alterations in collagen deposition during antifibrotic treatment [103]. Importantly, compared with FAPI PET, which reflects fibroblast activation and may be influenced by inflammatory or reparative processes, ⁶⁸Ga-CBP8 provides a more direct assessment of extracellular matrix deposition, potentially offering greater specificity for fibrotic remodeling. However, while FAPI imaging may be more sensitive to early fibroblast activity, CBP8 may better reflect structural collagen burden, highlighting the complementary rather than interchangeable roles of these tracers [85, 100, 104].

Another example, [⁶⁸Ga] Ga-Trivehexin (an αvβ6-integrin ligand), although primarily developed for oncologic applications, has demonstrated increased pulmonary uptake in isolated IPF case reports and small series [105]. These observations suggest possible applicability to fibrotic disease; however, diagnostic performance in ILD has not been established, and prospective validation is required. Similarly, preclinical studies of ¹⁸F-FPP-RGD₂ (an αvβ3 integrin tracer) detected increased integrin expression and fibrosis in bleomycin-induced rat models, with tracer uptake correlating with histologic severity [106]. These data indicate potential relevance to matrix remodeling in experimental fibrosis, but translation to human ILD remains unproven.

Another promising agent is the PDGFRβ-targeting radiotracer [^18^F]TZ-Z09591, which has been evaluated in animal models of lung injury and in ex vivo human tissue [107]. Increased uptake has been reported in areas of tissue remodeling, although background activity and contribution from non-fibrotic repair processes remain important limitations. Quantitative clinical validation is currently limited. Overall, while these tracers offer mechanistically complementary approaches to imaging fibrotic biology, their clinical utility, comparative performance, and prognostic value remain uncertain. Larger prospective human studies will be required before routine clinical use can be defined.

### Advantages of FAPI PET in pulmonary fibrosis

FAPI PET tracers have emerged as promising molecular imaging tools due to their high affinity for fibroblast activation protein (FAP), which is overexpressed in activated fibroblasts, particularly cancer-associated fibroblasts (CAFs) and fibrotic tissues [52, 108, 109]. This targeting enables selective visualization of fibroblast activity, supported by rapid internalization and relatively low nonspecific binding, resulting in often low background uptake [110, 111].

In the context of pulmonary fibrosis, a key advantage is the minimal physiological uptake in normal lung parenchyma, which enhances lesion detectability and improves image contrast compared to conventional imaging modalities [112, 113]. Favorable pharmacokinetics, including rapid renal clearance and low nonspecific uptake in most normal organs, further contribute to high imaging sensitivity and relatively low radiation exposure [114, 115]. FAPI PET imaging also allows rapid assessment of disease activity, with increased uptake in fibrotic lung tissue detectable as early as 15 min post-injection and optimal contrast typically achieved between 30 and 60 min [89]. Additionally, imaging can be performed without the need for fasting or glucose control, offering practical advantages in patients with metabolic conditions such as diabetes [116].

Although initially developed for oncology applications [117], FAPI tracers have demonstrated growing, albeit still limited, clinical potential in non-malignant fibrosis affecting organs such as the heart, liver, and kidney [61, 118, 119], as well as systemic fibrotic diseases [14, 110]. Preliminary studies have also reported correlations between FAPI uptake and FAP expression on histopathology, although validation remains limited by small sample sizes [93].

### Limitations and interpretation challenges

Despite these advantages, several important limitations and interpretation challenges must be considered, particularly in pulmonary fibrosis. A key limitation is the functional heterogeneity of FAP-expressing fibroblasts. Studies in oncology, extensive single-cell and functional work, have shown that cancer-associated fibroblasts (CAFs) comprise multiple subtypes, including myofibroblastic (myCAFs), inflammatory (iCAFs), and antigen-presenting (apCAFs) phenotypes, each with distinct biological roles [120, 121]. Similarly, single-cell RNA sequencing of fibrotic lung tissue has identified diverse fibroblast populations, such as myoCAF_FAP and iCAF_CXCL12 subtypes, with differing matrix-producing and inflammatory characteristics [122, 123]. However, the precise functional roles of these subpopulations in human disease remain incompletely understood, and direct translation from experimental models should be approached with caution. This heterogeneity has direct implications for molecular imaging. Current FAPI tracers bind broadly to FAP-expressing fibroblasts without distinguishing transient reparative cells from persistently activated profibrotic populations [124]. In idiopathic pulmonary fibrosis, although FAPI uptake correlates with lung function decline, it does not consistently reflect disease trajectory, suggesting that tracer uptake represents a composite of multiple biological processes rather than a single pathway [60]. Consequently, FAPI uptake should not be interpreted as a specific marker of irreversible fibrosis, as it may reflect transient tissue remodeling, chronic inflammation, or active fibrogenesis, leading to potential diagnostic ambiguity and false-positive findings in inflammatory conditions [14, 125–127].

Additional limitations arise from non-specific physiological uptake in certain organs. Hepatobiliary excretion results in prominent liver retention, while moderate uptake is observed in the pancreas and kidneys [68, 128]. The uterus may also demonstrate intense physiological accumulation, potentially obscuring adjacent pathology [112, 129]. Such background activity can complicate image interpretation and reduce specificity in certain clinical contexts [128]. To address these challenges, ongoing research is exploring advanced imaging strategies beyond FAP as a single molecular target. These include dual-tracer approaches, dynamic FAPI PET kinetic analysis [98, 130], integration with MRI, and correlation with spatial transcriptomic data [131, 132]. While these approaches may improve biological specificity, their clinical utility requires validation in prospective studies.

An important methodological consideration in pulmonary PET imaging is the influence of lung inflation and tissue density on quantitative measurements. SUV is affected by the air–tissue fraction within each voxel; thus, reduced aeration or increased density in fibrotic regions can lead to apparent increases in tracer uptake independent of biological activity—a phenomenon known as the tissue fraction effect [133]. Most PET studies, including FAPI imaging, rely on SUV-based metrics without correcting for lung density. Although CT attenuation correction is applied, it does not fully account for regional air content, and quantification errors may persist [134]. Indeed, increased lung density may artificially elevate tracer uptake before correction, with this effect diminishing after tissue fraction adjustment [133]. Therefore, elevated FAPI uptake in fibrotic lung should be interpreted cautiously, as it may reflect both true fibroblast activity and underlying density changes.

### Practical and clinical considerations

Practical limitations also influence the clinical implementation of FAPI PET. As a relatively novel imaging modality, the availability of FAPI tracers remains limited. Production using gallium-68 generators typically yields only a small number of patient doses per elution, contributing to higher per-dose costs [68]. Fluorine-18–labeled derivatives (e.g., ¹⁸F-AlF-FAPI) may offer improved scalability through cyclotron-based production [70, 76], although widespread adoption will depend on further validation and infrastructure development. From a clinical perspective, current evidence remains insufficient to support routine replacement of established imaging modalities such as ¹⁸F-FDG PET/CT. While FAPI PET may serve as a complementary tool in selected scenarios, its impact on cost-effectiveness, accessibility, and clinical workflows remains to be determined [135, 136].

## The future of FAPI radiotracers in pulmonary fibrosis

FAPI PET imaging in pulmonary fibrosis remains investigational; however, early preclinical and pilot clinical studies using tracers such as ⁶⁸Ga-FAPI-46 and ⁶⁸Ga-FAPI-04 demonstrate the feasibility of noninvasive visualization of fibroblast activation in vivo. These initial studies provide proof-of-concept for potential applications in early disease detection, monitoring, and therapeutic guidance, although robust clinical validation is still required.

### Early detection and stratification of disease activity

Early detection of fibrotic activity before irreversible structural damage is a major unmet need in pulmonary fibrosis. FAPI-based imaging targets fibroblast activation protein (FAP), which is upregulated in activated fibroblasts during early fibrogenesis [16, 137]. For example, in bleomycin-induced pulmonary fibrosis models, ⁶⁸Ga-FAPI PET demonstrated increased lung uptake as early as 7 days post-injury, correlating with histological fibrosis, whereas CT abnormalities appeared at later stages [85]. These findings suggest that FAPI PET may detect fibrogenic activity earlier than conventional imaging, although confirmation in human studies is required. Clinically, small pilot studies in patients with idiopathic pulmonary fibrosis (IPF) and other interstitial lung diseases (ILDs) have shown that uptake of tracers such as [¹⁸F] FAPI-74 correlates with disease severity and pulmonary function parameters [92]. Additionally, FAPI uptake has been reported to spatially correspond with fibrotic regions on CT, supporting its role as a marker of fibroblast activity rather than structural change [16, 138, 139]. While these studies suggest that FAPI PET may help distinguish active from chronic fibrosis, current evidence remains exploratory, and whether early FAPI positivity precedes irreversible fibrotic remodeling in humans remains to be established.

### Predicting disease progression and monitoring therapeutic response

FAPI PET/CT has shown promising preclinical results and emerging preliminary clinical signals for monitoring fibroblast activation during anti-fibrotic therapy. In human studies, elevated FAPI uptake correlates with respiratory impairment and radiographic severity in early IPF and systemic sclerosis-associated ILD [60, 95]. Although these correlations provide mechanistic insight, their prognostic and predictive value remains unproven. Robust clinical validation through larger prospective trials is essential before FAPI-PET can be considered an early-response biomarker for routine clinical use. At present, FAPI PET should be viewed as a promising investigational tool for enriching clinical trial cohorts, selecting patients with active fibrogenesis most likely to benefit from and demonstrate a response to novel anti-fibrotic agents.

### Guiding patient selection for anti-fibrotic therapy and accelerating drug development

By providing a molecular readout of fibroblast activation, FAPI PET may complement structural imaging in identifying patients most likely to benefit from anti-fibrotic therapy. In SSc-ILD, baseline uptake of ⁶⁸Ga-FAPI-04 has been reported to correlate with disease progression and clinical activity scores, partially independent of baseline CT findings and lung function [95]. Preclinical studies further support this concept. In bleomycin-induced fibrosis models, ⁶⁸Ga-FAPI uptake correlates with collagen deposition and decreases following treatment with antifibrotic agents such as pirfenidone [85]. These findings indicate that FAPI PET may serve as a sensitive imaging biomarker of treatment response. Incorporating FAPI PET into early-phase clinical trials has been proposed to provide imaging-based endpoints reflecting target engagement and biological activity. However, standardized imaging protocols, reproducibility studies, and multicenter validation are required before such applications can be implemented in clinical practice.

### Towards theranostics: targeting FAP+ fibroblasts

Several preclinical studies provide concrete examples of FAP-targeted theranostic strategies. Radioligand therapy using FAPI-based compounds, such as ¹⁷⁷Lu-FAPI-04, has demonstrated selective accumulation in fibrotic tissue, reduction of fibroblast activity, and improvement of organ function in animal models, including myocardial fibrosis [140]. In addition, various therapeutic isotopes (e.g., ¹⁷⁷Lu, ¹³¹I, Y-90) have been linked to FAPI tracers for combined imaging and targeted therapy in both oncologic and fibrotic contexts [108, 140]. Cell-based approaches also support this concept. FAP-targeted CAR-T and CAR-cTregs therapies have shown the ability to reduce fibrosis and improve tissue architecture in preclinical models, including pulmonary and cardiac fibrosis [141–143].

Despite these promising examples, significant challenges remain. Depletion of FAP⁺ fibroblasts may impair normal tissue repair, and issues related to dosimetry, safety, and off-target effects must be carefully addressed [144]. Furthermore, most available data are preclinical, and clinical experience in fibrotic lung disease is extremely limited. Overall, while FAPI-based theranostics represent a compelling translational direction, their clinical application in pulmonary fibrosis will require rigorous validation in human studies.

## Conclusion

FAPI PET imaging represents a promising investigational approach for visualizing active fibrogenesis in pulmonary fibrosis. Preliminary data suggest it may contribute to earlier detection, refined disease stratification, and assessment of therapy response. While challenges such as incomplete understanding of fibroblast heterogeneity, variability in FAP expression, and practical barriers to clinical adoption remain, these hurdles highlight the need for future research. At present, FAPI PET should be regarded as an investigational tool rather than a validated clinical modality in interstitial lung disease. Realizing its potential will require prospective multicenter trials, standardized imaging protocols, and correlation with clinical outcomes. Overall, FAPI-based molecular imaging has the potential to complement existing assessments, but its precise clinical role remains to be defined.

## Data Availability

Not applicable.
